# The Cause of Typhoid Fever in General, and the Cause of the Epidemic in Buffalo during March in Particular1Discussion of typhoid fever, before the Buffalo Academy of Medicine, April, 1894.— Part I.

**Published:** 1894-06

**Authors:** Charles Cary

**Affiliations:** 340 Delaware Avenue; Buffalo, N. Y.


Buffalo Medical and Surgical Journal

VOL. XXXIII. JUNE, 1894. No. 11.

### Original Communications.

### THE CAUSE OF TYPHOID FEVER IN GENERAL, AND THE CAUSE OF THE EPIDEMIC IN BUFFALO DURING MARCH IN PARTICULAR.[1]

BY CHARLES CARY, M. D., Buffalo, N. Y.

IN DISCUSSING the cause of typhoid fever at this time we want no originality or extravagant views, and with this thought in mind I have gathered from the most reliable of the authorities all the material which I here present, quoting from them without hesitation, so that statements herein made, unless my authority be questioned, are not open to cavil.

Typhoid fever can occur only from infection of the body with the typhoid bacilli, and is never incurred in any other way. Bad water, the products of decay and decomposition, tainted food, sewer gas, wet cellars and the like cannot of themselves produce the fever, unless the specific microörganism of typhoid be present. It is, however, none the less true that these surroundings do furnish a place for the accumulation of infection, or render persons susceptible by depressing their vitality. Furthermore, the introduction of the bacilli into the body does not necessarily produce the disease; for natural resistance to this, as to all disease, will often avoid infection. So that the vitality of the microörganism, and the vulnerability of the individual will determine the development of typhoid fever. These statements plainly point out the rational method of surveying the ground that I shall cover in my portion of the discussion—namely, the character of this so-called typhoid bacillus; second, its common means of introduction into the human body; and third, differing susceptibility of individuals as determined by climate, age, and the like.

1. Discussion of typhoid fever, before the Buffalo Academy of Medicine, April, 1894.—Part I.

Has it been proved that the bacillus is the sole cause of typhoid? It has been so demonstrated by the crucial bacteriological test. It is as fully proven as that the comma bacillus of Koch is the etiological factor in Asiatic cholera. It has been proven by satisfying the necessary two out of the three Koch laws; it is constantly present in every case of typhoid fever; it does not occur with other diseases as a harmless organism; it grows outside the body in a specific manner, but like cholera, leprosy and relapsing fever, the lower animals are not susceptible to the disease, and the final demonstration by inoculation is necessarily wanting.

I will not enter into the full description of this bacillus, but briefly, as pictured by Eberth, Koch, Gaffky and Frankel, it is one-third the size of a red blood corpuscle in length, one-third as thick as it is long, rounded at the extremities, and sometimes exhibiting at the center a shining rounded body, possibly a spore. It occurs singly or in filaments composed of a number of separate bacilli joined end to end. It is to be found in all the lesions of typhoid; it is chiefly found in the spleen, intestinal and mesental glands, enormous numbers of them are present in the passages from the eleventh to the seventeenth day of the disease, and they usually disappear after the twenty-second. Outside the body the bacilli will produce pure cultures on potato, gelatine, agar, blood serum, and bouillon. They grow rapidly in sterilized milk, and, in fact, there is scarcely any article of diet which does not form an excellent culture medium for these bacilli. They retain their vitality and perhaps multiply in damp soil. They may dry and in the form of dust be found in the air, but here they certainly do not increase. Sunlight depresses them; excessive heat destroys them; but they live for months in ice, as has been demonstrated by Prudden.

Finally, to complete this brief bacteriological description, these bacilli produce, as a result of their life process, various poisons, of which Breiger has described a ptomaine,—the typhotoxin; and Breiger and Frankel a toxalbumin, and Vaughn a ptomaine which when given to dogs produces vomiting and a rise of temperature. It is probable that these and analogous toxic products produce the constitutional symptoms in man of typhoid fever. Unfortunately, the detection of the typhoid bacillus cannot be made an ordinary clinical practice, as we at present obtain the tubercle bacillus, for it requires special experience and elaborate bacteriological equipment, and must, therefore, be delegated to experts.

To understand how this bacillus gains entrance into the human body, will be our next inquiry ; but we shall limit ourselves to statements regarding only the most common *viæ*. As has been stated, the infection is discharged mostly with the dejecta, and owing to its vitality it finds its way through our sewers or soil back to our water supply, to our milk, or perhaps as dust in the air, or still more rarely as food contamination. The disease is not contagious in the ordinary sense of the word, as there are no exhalations from the skin or lungs which can impart the disease. It may, however, be present in the material vomited or expectorated, or it may remain in the body after death, and it is only, therefore, in the careless handling of these materials and the dejecta that we can explain those cases of typhoid fever apparently due to contact. Our literature teems with instances of typhoid epidemics caused by water contamination, and the knowledge of this particular source of danger is not limited to the profession, nor even to the well-informed persons of an American community, but in an imperfect way it is realized in all grades of society.

As an instance, however, of this common cause of infection, I will recite one of the most remarkable epidemics of modern times, and one which is extensively quoted in standard works in medicine. It occurred in Plymouth, Pa., and was studied by Taylor and Shakespeare. In this instance a mountain stream, supplying a population of 8,000 people with drinking water, became infected from a single patient living close to the water's edge and some miles from the town. At the time of his illness with typhoid fever his attendants threw his evacuations on the ground and into the stream. As a result, 1,200 or nearly one-seventh of the entire population became infected, and these cases developed at the rate of from fifty to 100 a day ; about 100 persons perished.

Bronardel, in his paper before the Academy of Medicine of France, stated that the deaths in that country from typhoid fever aggregated 23,000 per annum, and he stated that the liability to this disease is directly in proportion to the defective water supply ; that when the water is improved the fever abates. He cited as an illustrative case Vienna, where the typhoid mortality was 200 per 100,000 so long as the inhabitants drank surface water, and it fell to ten per 100,000 when the water was improved. At Amiens the typhoid mortality of the military population was reduced from 111 per 10,000 to seven, by establishing artesian wells. At Rheims, typhoid fever was epidemic while the surface wells were used, and fell from forty-three per 10,000 to two, when they were abandoned. Van Fordor, of Buda-Pesth, reported an epidemic occurring in a Hungarian community of 34,000 people living on the side of a mountain, in which 1,228 persons were attacked, and ninety-three died. He states that 40 per cent. occurred in young persons. The preceding summer had been hot and dry. Late in October there was a copious rain-fall. Cases of enteric fever began to accumulate in the first week of November, the number became greatly increased in the second and third weeks, then rapidly declined, remaining, however, larger than usual. In the second week of the following February, after the melting of the surface snow, the epidemic again broke out, and continued for several weeks. Privy-wells were universal. Bacteriological examination of the mountain water supply showed it to be abundantly infected with bacilli having the essential characteristics of the typhoid bacillus.

Guimbretiere, of Boussy, reported the occurrence of a small epidemic of typhoid where all but two of the eighty-five persons affected had partaken of water from a well connected with a church in the village. On examination the water was found to be chemically impure, and to contain Eberth's bacilli. The two exceptional cases had nursed other persons suffering from the disease, and developed it after the subsidence of the epidemic. The source of the contamination of the well was obscure, but the church itself occupied the site of an old cemetery. The recital of these epidemics will suffice to illustrate infection from water contamination. The examples might be multiplied indefinitely.

Infection by milk is by no means uncommon, and ten years ago the *British Medical Journal* showed that up to that date fifty epidemics of typhoid fever had been traced to this cause. This contamination of milk need not necessarily be the result of premeditated adulteration or dilution, but may take place from the legitimate use of water for ordinary cleanliness; for, be it understood, milk contamination commonly occurs from infected water. Sedgwick and Chapin detail an analysis of an epidemic of enteric fever in the City of Springfield, Mass., and Sedgwick reports the results of an epidemic in Sommerville, Mass. The number of cases discovered and investigated in the first of these two, was 150, and the deaths twenty-five. The milk was contaminated by placing the cans submerged in a well which contained dejecta from cases of enteric fever. The cans were not so hermetically sealed as to prevent the entrance of water. The milk was placed in the well for the purpose of keeping it cold. The other epidemic occurred among the customers of one milkman, and the investigation disclosed the fact that the son of this man handled and delivered the milk while suffering from an attack of enteric fever. The disease was at first unrecognized, and he continued his work until thirty-two persons became infected.

Mitchell reports twelve cases in persons who drank milk from a common source. There was typhoid fever in the dairyman's family, and those who milked the cows nursed also the typhoid patient.

Caton, of Liverpool, has directed attention to the possible dissemination of typhoid fever from the use of liquid manure obtained from cess-pools, in the cultivation of lettuce, celery and allied vegetables.

Henry E. Armstrong, medical officer of health of New Castle-on-Tyne, in his article on the different diseases communicated to man by milk, states that epidemics from this source appear usually as a sudden outburst, and that with the modern method of reporting to the Board of Health all cases of infectious diseases there is at present no difficulty in fixing the source of contamination to milk.

Finally, not in the form of an epidemic outburst, but simply as isolated cases, filthy clothing may be the carrying medium, and was the cause assigned to the continuance of typhoid fever in certain military quarters in Germany. Among contributing causes climate exerts a certain influence. The disease occurs in all climates, though it is most abundant in the temperate zone. It is not uncommonly found in the tropics, is common in Iceland, Norway and Finland. Season, again, exerts a marked influence and though constantly present with us it is far more frequent in the late summer and autumnal months. It is more common after hot and dry summers, and Pettinhoffer and Buhl say that the disease was most common when the water was low, it being believed that when the water level was low, the greater must be the solid matter suspended in it. Also dust of dry seasons may disseminate the germs. It is also probable that when the dry season breaks up, the changeable weather brings with it intestinal catarrhal troubles, which favor the lodgement of the germs in the intestine. Sex does not influence the disease, but age does in a marked manner. This is the disease of youth and adult life, the greatest susceptibility being between the ages of fifteen and twenty-five years.

Lastly, there is, as would appear, an inherited predisposition, for some families certainly contract the disease with greater readiness than others. This cause is probably anatomical.

To summarize, then, the causes of typhoid fever, the one only true existing cause is the typhoid bacillus, which finds its way into the body through the mouth, with the drinking water most commonly, more rarely with milk or dust or food contamination; in still more rare instances by the careless handling of typhoid material, and in perhaps one case which has come under my observation, by direct transfusion. The contributing causes are age, climate, season, soil, individual and inherited susceptibility, unclean habits and intestinal disturbance.

In conclusion, I am asked to assign the cause of this particular epidemic—our epidemic—of 500 cases in very nearly one month. This, like any other, must be caused by the typhoid bacillus. The inquiries into the mode of its conveyance and wide distribution in our city have not been completed, though the investigation is still being carried on, so that we may hope to profit fully by our hard experience; still it may be said that the epidemic was without much question caused by temporary contamination of our water. Spread as these cases were rather evenly throughout the city, it is not probable that the cause could be milk or food supply, for there is no one source from which they are derived. Soil and air have never been the cause of an epidemic of this character, whereas water is the most common of all causes, and with this knowledge we turn to the examination of this commodity and find our suspicions warranted, and proof almost fixes the cause here. The Bird Island Inlet has been one of our sources of water for many years. The Bird Island Inlet has been the source from which we have drawn water frequently through this Winter. When the water in the river was low, and just preceding the development of our epidemic, then also was the Bird Island Inlet open and our supply was taken from this source.

In confirmation, our City Bacteriologist, after careful examination, has reported the abundant contamination of the Niagara river water at this inlet, and has found Eberth's typhoid bacillus in it.

Note.—The usual water supply is taken through a tunnel from the crib in the center of the river. The Bird Island Inlet is on the east bank of Niagara River and is separated from the Erie Canal by the canal feeder; both of these waters are foul.

340 Delaware Avenue.

